# Roles of acid-extruding ion transporters in regulation of breast cancer cell growth in a 3-dimensional microenvironment

**DOI:** 10.1186/s12943-016-0528-0

**Published:** 2016-06-06

**Authors:** Anne Poder Andersen, Mette Flinck, Eva Kjer Oernbo, Nis Borbye Pedersen, Birgitte Martine Viuff, Stine Falsig Pedersen

**Affiliations:** Department of Biology, Section for Cell Biology and Physiology, Faculty of Science, University of Copenhagen, Universitetsparken 13, DK-2100 Copenhagen, Denmark; Department of Veterinary Disease Biology, Section for Molecular Disease Biology, Faculty of Health and Medical Sciences, University of Copenhagen, Strandboulevarden 49, DK-2100 Copenhagen, Denmark

**Keywords:** Tumor microenvironment, Acid–base transport, NHE1, NBCn1, MCT1, MCT4

## Abstract

**Background:**

The 3-dimensional (3D) microenvironment of breast carcinomas is characterized by profoundly altered pH homeostasis, reflecting increased metabolic acid production and a confined extracellular space characterized by poor diffusion, yet the relative contributions of specific pH-regulatory transporters to 3D growth are poorly understood. The aim of this work was to determine how 3D spheroid growth of breast cancer cells impacts the expression and spatial organization of major acid extruding proteins, and how these proteins in turn are required for spheroid growth.

**Methods:**

MCF-7 (Luminal-A) and MDA-MB-231 (Triple-negative) human breast cancer cells were grown as ~700-950 μm diameter spheroids, which were subjected to Western blotting for relevant transporters (2- and 3D growth), quantitative immunohistochemical analysis, and spheroid growth assays. Individual transporter contributions were assessed (i) pharmacologically, (ii) by stable shRNA- and transient siRNA-mediated knockdown, and (iii) by CRISPR/Cas9 knockout.

**Results:**

In MCF-7 spheroids, expression of the lactate-H^+^ cotransporter MCT1 (SLC16A1) increased from the spheroid periphery to its core, the Na^+^,HCO_3_^−^ cotransporter NBCn1 (SLC4A7) was most highly expressed at the periphery, and the Na^+^/H^+^ exchanger NHE1 (SLC9A1) and MCT4 (SLC16A3) were evenly distributed. A similar pattern was seen in MDA-MB-231 spheroids, except that these cells do not express MCT1. The relative total expression of NBCn1 and NHE1 was decreased in 3D compared to 2D, while that of MCT1 and MCT4 was unaltered. Inhibition of MCT1 (AR-C155858) attenuated MCF-7 spheroid growth and this was exacerbated by addition of S0859, an inhibitor of Na^+^,HCO_3_^−^ cotransporters and MCTs. The pharmacological data was recapitulated by stable knockdown of MCT1 or NBCn1, whereas knockdown of MCT4 had no effect. CRISPR/Cas9 knockout of NHE1, but neither partial NHE1 knockdown nor the NHE1 inhibitor cariporide, inhibited MCF-7 spheroid growth. In contrast, growth of MDA-MB-231 spheroids was inhibited by stable or transient NHE1 knockdown and by NHE1 knockout, but not by knockdown of NBCn1 or MCT4.

**Conclusions:**

This work demonstrates the distinct expression and localization patterns of four major acid-extruding transporters in 3D spheroids of human breast cancer cells and reveals that 3D growth is dependent on these transporters in a cell type-dependent manner, with potentially important implications for breast cancer therapy.

**Electronic supplementary material:**

The online version of this article (doi:10.1186/s12943-016-0528-0) contains supplementary material, which is available to authorized users.

## Background

Cells in a solid tumor function in an environment fundamentally different from that of cells in normal tissues, which profoundly affects the gene expression profile and functional properties of the cancer cells. This has been demonstrated both for physico-chemical conditions in the tumor microenvironment [1] and for the 3-dimensional (3D) tumor architecture [2, 3]. Comparisons of 2D- and 3D culture of cancer cells have demonstrated that the phenotype of 3D spheroids much more closely mimic that of in vivo tumors [4, 5]. The specific tumor microenvironment conditions differ between cancer forms, but generally comprise hypoxia and/or anoxia, reduced glucose and ATP levels, elevated extracellular lactate levels, and acidic extracellular pH (pH_e_) [6–8]. In addition to hypoxia, the *Warburg effect*, i.e. a shift from oxidative phosphorylation towards glycolysis even in the presence of sufficient oxygen, is characteristic of most cancer cells, favoring predominant use of glycolytic metabolism [9]. Elevated rates of glycolysis and ATP hydrolysis cause the highly proliferative and anabolic cancer cells to produce more acid than normal cells [7]. As a slightly alkaline intracellular pH (pH_i_) value is a prerequisite for growth, proliferation, survival and motility [10, 11], cancer cells must initiate strategies to circumvent intracellular acidification.

Mediators of increased acid extrusion in tumor cells include the Na^+^/H^+^ exchanger NHE1 (SLC9A1), the Na^+^,HCO_3_^−^ cotransporter NBCn1 (SLC4A7), the lactate,H^+^ cotransporters of the monocarboxylate transporter family, MCT1 and MCT4 (SLC16A1 and −3), and, in some cells, V-type H^+^-ATPases [8, 12–14]. We and others have previously demonstrated upregulation of these transporters in human cancers [8, 15–18]. Specifically, we recently reported the strong upregulation of NBCn1 by the major breast cancer oncogene ErbB2 [12, 19], and genome-wide association studies (GWAS) have consistently demonstrated association of a SNP in the NBCn1 3’ untranslated region with increased risk of breast cancer [20]. In vivo studies demonstrated roles for MCTs and NHE1 [13, 21] and our own recent work identified an important role for NBCn1 [22] in tumor growth.

Altered pH homeostasis plays central roles in many aspects of tumor progression [6–8, 10], and studies in 2D monoculture have implicated specific pH-regulatory ion transporters in control of e.g. metabolism, motility, and chemotherapy resistance in a wide range of cancer cell lines [12, 23–25]. However, regulation of pH_i_ in solid tumors differs profoundly from that in 2D culture, and major regional differences and dynamic changes in the roles of pH-regulatory proteins as the tumor grows can be envisaged. Elegant studies of 3D tumor spheroids have demonstrated the predicted gradient of increasing extracellular acidity toward the spheroid core [26], but detailed studies of the spatial organization of individual pH-regulatory transporters and of their contributions to spheroid growth are lacking.

The aim of the present study was to provide, for the first time, a detailed analysis of the impact of 3D growth on expression and localization of pH-regulatory ion transporters, and determine how these transporters in turn impact on 3D growth of breast cancer cells. We show that 3D spheroid growth impacts on the relative expression of acid-extruding transporters, and that NHE1, NBCn1, MCT1 and MCT4 exhibit distinct spatial organization within MCF-7 and MDA-MB-231 breast cancer cell spheroids. Furthermore, we show that 3D growth of MCF-7 cells is predominantly dependent on NBCn1 and MCT1, while that of MDA-MB-231 cells is highly dependent on NHE1. Thus, we demonstrate that acid-extruding transporters exhibit distinct localization patterns in breast cancer spheroids and are important for their growth in a cell-type dependent manner, with potentially important implications for breast cancer therapy.^1^

## Methods

### Antibodies and reagents

The following antibodies were purchased from Cell Signaling: Ezrin/Radixin/Moesin (#3142), PARP (#9542) and cleaved PARP [Asp214] (#5625). NHE1 (#sc-136239), MCT4 (#sc-50329) and ZO-1 (#sc-10804) were from Santa Cruz Biotechnology, and MCT1 (#AB3538P) from Millipore. E-cadherin (#610181) and p150 [Glued] (#610473) were from BD Transduction Laboratories and CAIX (M75, [28]) was from BioScience, Slovakia. The NBCn1 antibody was a kind gift from Jeppe Praetorius, Aarhus University, Denmark, and the polyclonal NHE1 antibody (Xb-17) was a kind gift from Mark Musch, University of Chicago, IL. Antibody against the S703-phosphorylated NHE1 was from DSTT, University of Dundee, Ireland. β-actin (#A5441), anti-Mouse (#A1293) and anti-Rabbit (#A3937) alkaline phosphatase-conjugated secondary antibodies were from Sigma-Aldrich. Anti-mouse (#P0447) and anti-rabbit (#P0448) Horseradish Peroxidase (HRP)-conjugated secondary antibodies were from Dako. Cariporide and S0859 were kind gifts from Sanofi-Aventis. AR-C155858 was acquired from AdooQ BioScience, Irvine, CA, USA. The Hydroxyprobe-1 kit including Pimonidazole and anti-pimonidazole mouse monoclonal antibody was purchased from Hydroxyprobe, Inc., Burlington, MA, USA.

### Cell lines and general cell culture

MCF-7 (a kind gift from Dr. Lone Ronnov-Jessen, University of Copenhagen) and MDA-MB-231 cells (a kind gift from Dr. Marie Kveiborg, University of Copenhagen) were grown in DMEM 1885-medium (incl. NaHCO_3_^−^) (Panum 22-2-24, #015) supplemented with 6 % FBS for MCF-7 cells and 10 % FBS for MDA-MB-231 cells (Gibco, #10 106–177), 1 % Pen/Strep (Invitrogen, #15140-148), and 1 % MEM Non-Essential Amino Acids 100× (Gibco/Invitrogen, #11140-035). Cell cultures were discarded when they reached passage 22. For growth of stabile shRNA knock down cell lines of MCF-7 and MDA-MB-231, the medium was additionally supplemented with 1 μg/mL Puromycin (Gibco, #A11138-02). Cells were grown at 37 °C, 95 % humidity, 5 % CO_2_ and passaged when a confluence of 70-80 % was reached.

### 3D spheroid formation and corresponding 2D cultures

1000 MCF-7 or MDA-MB-231 cells were seeded per well in round bottomed, ultra-low attachment 96-well plates (Corning, #7007) in 200 μL DMEM 1885-medium. MDA-MB-231 cells were centrifuged for 15 min at 750 RCF at 4 °C and both cell types were grown for the indicated number of days (4–9) at 37 °C with 95 % humidity and 5 % CO_2_. Medium for spheroids of MDA-MB-231 cells was additionally supplemented with 0.24 μg/μL GelTrex LDEV-Free Reduced Growth Factor Basement Membrane Matrix (Gibco/Invitrogen, #A1413202). For some experiments, corresponding 2D monolayer cultures were performed: 500,000 MCF-7 cells/mL or 400,000 MDA-MB-231 cells/mL were seeded in 10 cm Petri dishes and grown for 4 days at 37 °C with 95 % humidity and 5 % CO_2_.

### Spheroid growth assays

Compact spheroids formed two days after the cells were seeded in round bottomed, ultra-low attachment 96-well plates and for the indicated experiments, Cariporide (10 μM), S0859 (50 μM), AR-C 155858 (20 μM) and corresponding vehicle were added as indicated and the spheroids were grown for additionally seven days. 100 or 150 μL medium (incl. inhibitors, when indicated for the experiment) was exchanged every second day. Light microscopic (Nikon, Japan or Leica MZ16, Germany) images of the spheroids at 10× magnification were acquired on the day of addition of inhibitors (day 2) and on day 4, 7, and 9. To quantify spheroid growth, spheroid diameters were measured using ImageJ software. In Figs. 5d and 6e, spheroid area was measured using the freehand-drawing function of ImageJ. Each data point thus represents the mean of measurements on 3–12 spheroids per condition (except for MDA-MB-231 transient knockdown and CRISPR/Cas9 knockout experiments, where in a few cases only 2 spheroids per condition could be measured).

### Lentiviral knockdown of NHE1, NBCn1, MCT1 and MCT4

The following Mission shRNA bacterial glycerol stocks were purchased from Sigma-Aldrich: NHE1 (NM_003047), TRCN0000044649; NBCn1 (NM_003615), TRCN 0000043159; MCT1 (NM_003051), TRCN 0000038340; MCT4 (NM_004207). Envelope plasmid pMD2.G and packaging plasmids pMDLg/pRRE and pRSV-Rev, and pLKO.1 empty vector plasmid were a kind gift from Jacob B. Hansen, University of Copenhagen. Overnight bacterial cultures were cultivated in LB medium incl. ampicillin (Sigma-Aldrich, #A9518) for selection and plasmid DNA purification was done by using a Nucleobond Xtra Midi EF kit (Macherey-Nagel GmbH & Co. KIG, Germany) according to manufacturer’s instructions.

#### Transfection of HEK293T cells

HEK293T cells were seeded in 6 cm Petri dishes and grown to approx. 70 % confluence (one dish for each plasmid DNA). 2.75 μg purified plasmid DNA encoding shRNA sequences to target NHE1 (NM_003047), NBCn1 (NM_003615), MCT1 (NM_003051), and MCT4 (NM_004207), respectively, and a pLKO.1 empty vector plasmid was mixed with 0.75 μg pMD2.G, 0.75 μg pMDLg/pRRE, 0.75 μg pRSV-Rev, 12.5 μl FuGENE HD Transfection reagent (Promega, #E2311) and DMEM basal medium was added up to 250 μl. The mixture incubated 15 min at room temperature (RT) and was added to the HEK293T cells in DMEM-1885 medium without Pen/Strep.

#### Transduction of MCF-7 and MDA-MB-231 cells

The next day, medium on the HEK293T cells was refreshed and the day after virus-containing medium was harvested and 1.5 mL was sterile filtered through 0.45 μm filters and added to ~20 % confluent MCF-7 and MDA-MB-231 cell cultures along with 1.5 mL medium and 4.5 μl polybrene (5 mg/mL; Sigma, #S2667). To assure efficient transduction a parallel dish of non-transduced MCF-7 and MDA-MB-231 cells was included and was killed 100 % by Puromycin. Fresh medium was added to the HEK293T cells and the same procedure for harvesting of virus-containing medium was repeated the next day. When the transduced MCF-7 and MDA-MB-231 cells had grown for 24 h, they were reseeded and selection with 1 μg/mL Puromycin (Gibco, #A11138-02) started. Medium was changed every 2–3 days to assure complete selection pressure and the cells were split when needed.

### CRISPR/Cas9 gene editing

MDA-MB-231 (1 x 10^6^) and MCF-7 (2 x 10^6^) cells were transfected with 1 or 2 μg pX458 plasmid targeting exon 1 of *SLC9A1* using Amaxa nucleofection (Lonza) with the V-kit according to manufacturer’s guidelines. Transfectants were cloned by limiting dilution and screened using immunoblotting against NHE1. Mutations in *SLC9A1* were confirmed by PCR using 5’-CTGTGGCCTCTCTCCACATC-3’ and 5’-TCGGAGCAAACGGGACTTAC-3’ followed by sequencing. A detailed description of the CRISPR/Cas9 clones is forthcoming in a manuscript currently in preparation.

### Transient knockdown

MDA-MB-231 and MCF-7 cells were seeded in 6-well plates and grown to approximately 70 % confluency. MDA-MB-231 cells were treated with 100 nM siNHE1 (ON-TARGET SMARTpool, Thermo Scientific). Mock siRNA (Sense sequence: 5′-AGGUAGUGUAAUCGCCUUGUU-3′, Eurofins MWG Operon, Ebersberg, Germany) at corresponding concentrations was included as a control. Transfections were performed using Lipofectamine 2000 (Life Technologies, #11668-019) in DMEM 1885 medium without Pen/Strep. The medium was replaced with normal growth medium after 24 h, and spheroid formation was initiated after another 24 h by seeding the transfected cells in round-bottomed ultralow attachment 96-well plates (Corning, #7007) as described above.

### Immunoblotting

#### 2D culture

Cells were grown to 70–90 % confluency in 10 cm Petri dishes, washed in ice-cold PBS and lysed in lysis buffer (1 % SDS, 10 mM Tris–HCl, 1 mM NaVO_3_, pH 7.5, heated to 95 °C). The cell lysates were homogenised by sonication (PowerMED, Portland, Maine) and centrifuged (Micromax RF, Thermo) for 5 min at 20,000 g at 4 °C to remove cell debris.

#### 3D culture

Spheroids were collected in Eppendorf tubes, washed once in 1 mL ice-cold PBS and lysed in lysis buffer (1 % SDS, 10 mM Tris–HCl, 1 mM NaVO_3_, pH 7.5, heated to 95 °C) for ~10 min at RT with intervals of vigorous vortexing. After this, the procedure for homogenization and removal of cell debris described for 2D culture was followed.

#### SDS-PAGE and immunoblotting of 2D and 3D cultures

Lysate protein content was determined (DC Protein Assay kit, Bio-Rad), equalized with ddH_2_O, and NuPAGE LDS 4x Sample Buffer (Invitrogen, #NP0007) and Dithiothreitol (DTT) added. Proteins were separated by SDS-PAGE under denaturing and reducing conditions using precast NuPAGE 10 % Bis-Tris gels (NOVEX by Life Technologies, #NP0302BOX) and NuPAGE MOPS SDS Running Buffer (NOVEX by Life Technologies, #NP0001) or Criterion TGX 10 % gels (BioRad, #567-1034 (18 wells) or #567-1035 (26 wells)) and Tris/Glycine SDS buffer (BioRad, #161-0732), and Benchmark protein ladder (Invitrogen, #10747-012). Separated proteins were transferred to a nitrocellulose membrane (Invitrogen, #LC2000) using NuPAGE Transfer Buffer (NOVEX by Life Technologies, #NP0006) or to Transblot Turbo 0.2 μm nitrocellulose membranes (BioRad, #170-4159). Membranes were stained with Ponceau S (Sigma-Aldrich, #P7170-1 L), blocked in blocking buffer (5 % nonfat dry milk in TBST (0.01 M Tris/HCl, 0.15 M NaCl, 0.1 % Tween 20)) for 1 h at 37 °C, incubated overnight at 4 °C with primary antibodies diluted in blocking buffer, washed extensively in TBST, incubated with alkaline phosphatase- or HRP-conjugated secondary antibodies diluted in blocking buffer, washed in TBST, and developed using BCIP/NBT Phosphatase Substrate (KPL, #50-81-08) or by chemiluminescence using Pierce ECL Western Blotting Substrate (Thermo Scientific, #32209), respectively. The membranes were scanned and band intensity quantified using UN-SCAN-IT 6.1 (Silk Scientific). Protein expression was normalized to that of the corresponding control.

### Spheroid fixation and immunohistochemistry

Spheroids were collected in Eppendorf tubes, washed once in 1 mL ice-cold PBS, fixed in 10 % PBS buffered formalin for 18–24 h at RT where after they were washed twice in PBS, resuspended in a small amount of PBS and injected into a drop of 60 °C warm 2 % agarose solution (Bactoagar; BD, #214050). For pimonidazole labeling, spheroids were pretreated with 100 μm pimonidazole 2 h before fixation. Once the agarose had solidified at 5 °C for approx. 5 min, the drop was transferred to an embedding cassette, embedded in paraffin and sliced using a microtome (ZEISS, MIKROM HM 450). Paraffin sections of spheroids were deparaffinized by passing them through decreasing alcohol concentrations for 3 min each: Xylene (6 min), 1:1 mixture of xylene and 99.9 % ethanol, 99.9 % ethanol, 96 % ethanol, 70 % ethanol, 50 % ethanol. The sections were washed in cold water, placed in citrate buffer (0.21 % citric acid monohydrate (Sigma-Aldrich, #C1909) in ddH_2_O, pH 6) and irradiated in a domestic microwave oven for 3 × 5 min with 1 min interval. After cooling to RT, the sections were encircled using a pap pen, washed once in PBS before Tris-glycine (0.1 M glycine (AppliChem, #A3707) in ddH_2_O, pH 7.4) was added for 15 min. After washing in PBS, the sections were blocked in 5 % BSA in PBST (0.1 % Tween) for 30 min–1 h RT and incubated overnight at 4 °C with primary antibodies diluted in 1 % BSA in PBST. The sections were washed in PBST (3 × 5 min), incubated with 1 % BSA in PBST for 15 min and afterwards with fluorophore-conjugated secondary antibodies diluted 1:600 in 1 % BSA in PBST for 30 min RT. The sections were washed in PBST (4 × 5 min) including DAPI (1:1000), mounted with N-propyl gallate and sealed with nail polish. The fluorescence-labeled proteins were visualized using an Olympus BX63 epifluorescence microscope (40× or 10× objective). Subsequent image adjustments (overlays and intensity only) were performed in Adobe Photoshop CS6. Essentially no labeling was detectable in absence of primary antibodies (not shown). To quantify the intensity of labeling across the spheroids, mean pixel intensity profiles through the spheroids were calculated using ImageJ software, based on rectangular Regions of Interest (ROIs) of 50 or 100 μm widths, going from the spheroid periphery to core (two-three independent biological replicates per transporter/condition). For each intensity profile, the x-axis was set to start at zero and x-values were normalized to the largest x-value resulting in an x-axis ranging from 0–1. The intensity values (y-axis) were likewise normalized to the largest intensity, generating a y-axis ranging from 0–1. The resulting intensity profiles were plotted in a scatter plot and LOWESS curves with 20 points in the smoothing window were drawn using GraphPad PRISM 6 in order to better visualize trends of the data.

### Statistical analysis

All data are shown as representative images or as means with standard error of means (SEM) error bars. A two-tailed, paired (when applicable, otherwise unpaired) Student’s t-test was used to test for statistically significant difference in means between two groups. One-way analysis of variance (ANOVA) followed by Tukey’s or Dunnett’s multiple comparisons post-test was used to test for statistically significant differences when there were more than two groups. (*), *, **, ***, and, **** denote *p* < 0.1, *p* < 0.05, *p* < 0.01, *p* < 0.001 and *p* < 0.0001, respectively.

## Results

### MCF-7 spheroids exhibit polarized growth, lumen formation, a gradient of hypoxia, and non-homogenous distribution of pH-regulatory transporters

**Fig. 1:**
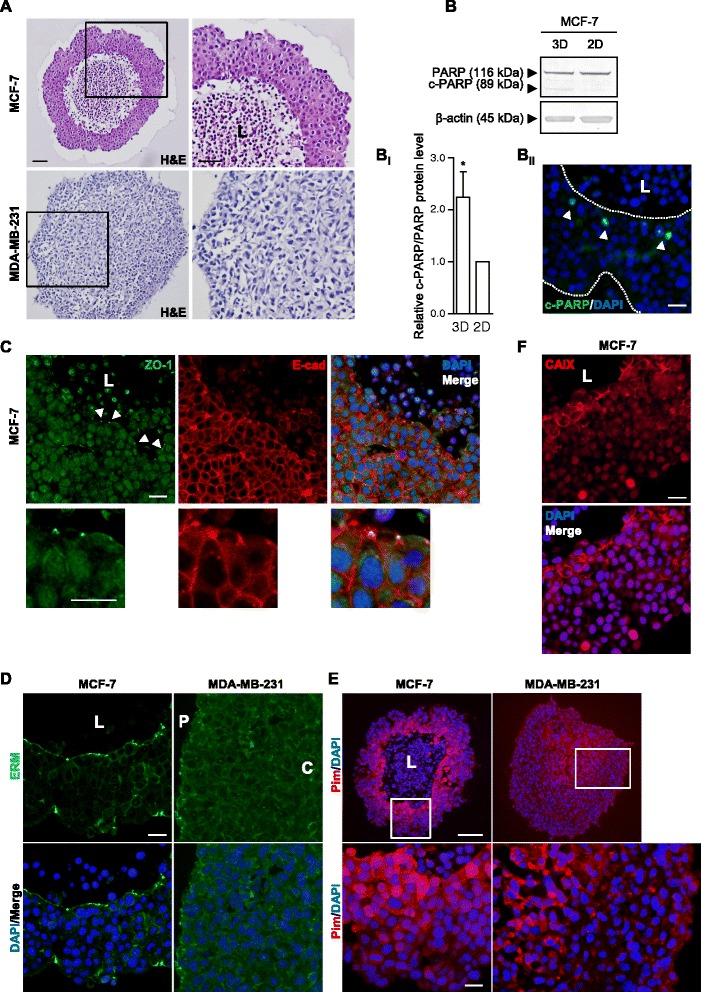
Characterization of MCF-7 and MDA-MB-231 spheroids. Sections of MCF-7 and MDA-MB-231 spheroids that were grown for 9 days (**a**, **b**_**ii**_, **c**, **d**, **e** and **f**) followed by PFA fixation, embedding, and histological and immunohistochemical analysis (IHC). **a**: Hematoxylin and eosin (H&E) staining. **b**: MCF-7 spheroids (3D) and 2D cultures were grown for 4 days in parallel followed by lysis and Western blotting with PARP antibodies. Top panel shows representative Western blots, while panel **b**_**i**_ shows quantifications of band intensities normalized to that of corresponding 2D culture. Error bars denote SEM. 8 n. A two-tailed, paired Student’s t-test was used to test for statistically significant difference in means between the two groups. * indicates *p* < 0.05. Panel **b**_**ii**_ shows a section of a MCF-7 spheroid stained with antibodies recognizing cleaved PARP (c-PARP) only. Dashed lines indicate spheroid boundaries and arrowheads indicate c-PARP positive nuclei. Scalebar: 20 μm. **c**: ZO-1 and E-cadherin staining of MCF-7 spheroids. Arrowheads indicate ZO-1 puncta. Scalebars: 20 μm. **d**: Ezrin/Radixin/Moesin (ERM) staining. Scalebar: 20 μm. **e** and **f**: Pimonidazole (Pim) and CA IX staining was used to detect hypoxic regions. Scalebars: 100 μm and 20 μm, respectively. All images are representative of 4–5 n, except for data in **d**-right and **e**, which represent 3n. L, P and C: indicate lumen, periphery and core, respectively, of spheroids

While they do not form single cell-layered acini [29], MCF-7 breast cancer cell spheroids can polarize [30]. To characterize their polarization properties, MCF-7 spheroids were fixed and processed for histology. Hematoxylin-eosin (HE) staining illustrated the formation of a core of cells with pyknotic nuclei (Fig.1a, top), in contrast to the spheroids formed by the more poorly differentiated MDA-MB-231 breast cancer cells, which are more loosely organized and completely lack a central lumen (Fig. 1a, bottom). The lumen formation could involve apoptotic [31] or necrotic [5] cell death in the spheroid core. To evaluate apoptosis induction in the spheroids, we assessed PARP-1 cleavage during spheroid growth and in 3D relative to 2D culture. PARP-1 cleavage was increased in 3D growth relative to 2D conditions (Fig. 1bi) and during the course of spheroid growth (Additional file 1: Figure S1A), and was most prominent in cells adjacent to the spheroid lumen (Fig. 1bii and Additional file 1: S1Bi). In contrast, PARP-1 cleavage was essentially undetectable in the MDA-MB-231 spheroids (Additional file 1: Figure S1Bii). Expression patterns of E-cadherin and the tight junction protein ZO-1 were analyzed to evaluate polarization (Fig. 1c). ZO-1 puncta indicative of tight junctions, were visible just below the luminal surface, while E-cadherin staining was essentially lacking at apical membranes facing the lumen, consistent with its prominent lateral localization. Ezrin/radixin/moesin (ERM) protein staining confirmed the formation of an apical surface toward the spheroid lumen of MCF-7 cells, while MDA-MB-231 cells lacked a central lumen and showed even ERM protein distribution throughout the spheroids (Fig. 1d). Collectively, these results show that the MCF-7 spheroids are polarized with an apical lumen, while this is not seen in the more malignant and less differentiated MDA-MB-231 cells. Pimonidazole staining (Fig. 1e) demonstrated the existence of an inwardly directed gradient of hypoxia in both cell types, confirmed by staining for the highly hypoxia-inducible carbonic anhydrase IX (CAIX) in MCF-7 cell spheroids (Fig. 1f). Further corroborating the association of CAIX expression with induction of a hypoxic inner region of the spheroids, CAIX protein was undetectable (MCF-7) or weakly expressed (MDA-MB-231) in 2D and at day 4 of spheroid growth but strongly induced at day 9 in spheroids of both cell types (Additional file 1: Figure S1Ci-ii).

**Fig. 2:**
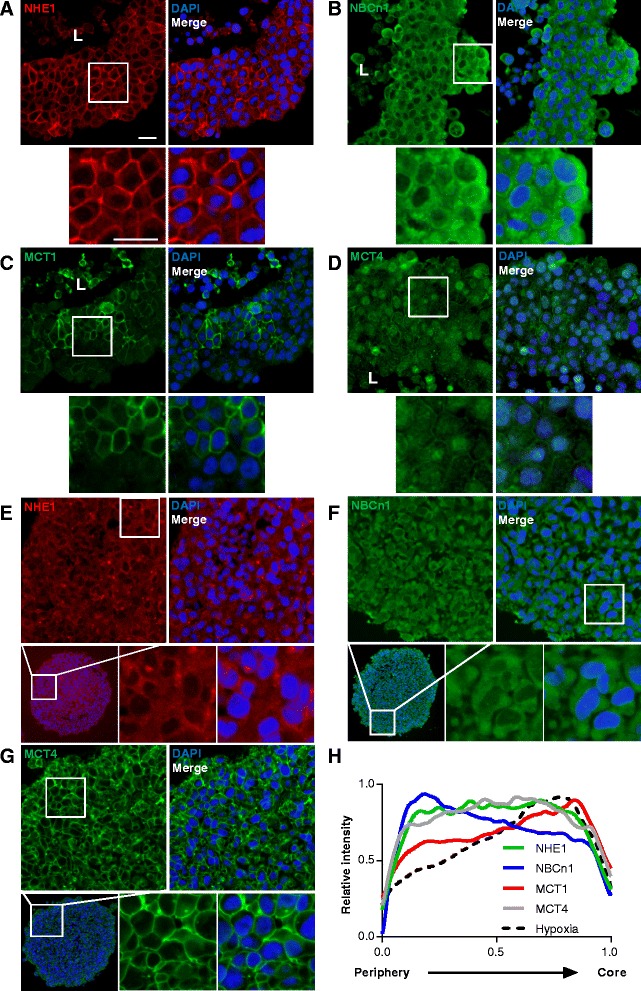
Localization profiles of NHE1, NBCn1, MCT1 and MCT4 in MCF-7 and MDA-MB-231 spheroids. Sections of MCF-7 (A-D) and MDA-MB-231 (E-G) spheroids that were grown for 9 days followed by PFA fixation, embedding, and immunohistochemical analysis (IHC). **a** and **e**: NHE1 staining. **b** and **f**: NBCn1 staining. **c**: MCT1 staining. **d** and **g**: MCT4 staining. Images are representative of 3–5 n. Scalebars: 20 μm. L: indicates lumen of spheroid. **h**: Relative distribution of the transporters NHE1, NBCn1, MCT1 and MCT4 from the periphery towards the core (across the viable region) of MCF-7 spheroids. Based on two mean pixel intensity profiles per spheroid on three spheroids (only two for MCT1) from independent biological replicates per transporter/antibody (For details, see Methods)

The core and periphery of cancer cell spheroids have been shown to exhibit differential pH_i_-regulatory properties [26]. To determine whether this involves differential expression of individual transporters in different regions of the spheroid, MCF-7 spheroids were sectioned and stained for NHE1, NBCn1, MCT1 and MCT4 (Fig. 2a-d). In MCF-7 spheroids, NHE1 and MCT4 localized homogenously to cell membranes (and, in the case of MCT4, also in intracellular compartment(s), see Discussion) throughout the spheroids. In contrast, NBCn1 was most highly expressed at the spheroid periphery, with staining seen both in membranes and intracellularly, and MCT1 showed strong membrane localization and a clear gradient of increasing expression toward the spheroid core. For comparison, MDA-MB-231 cells (which do not express MCT1 due to aberrant promoter methylation [32]) were stained for NHE1, NBCn1, and MCT4 (Fig. 2e–g). The most remarkable difference from the pattern observed in MCF-7 spheroids was that MCT4 expression was much more strongly membrane-localized in MDA-MB-231 spheroids (Fig. 2g).

**Fig. 3:**
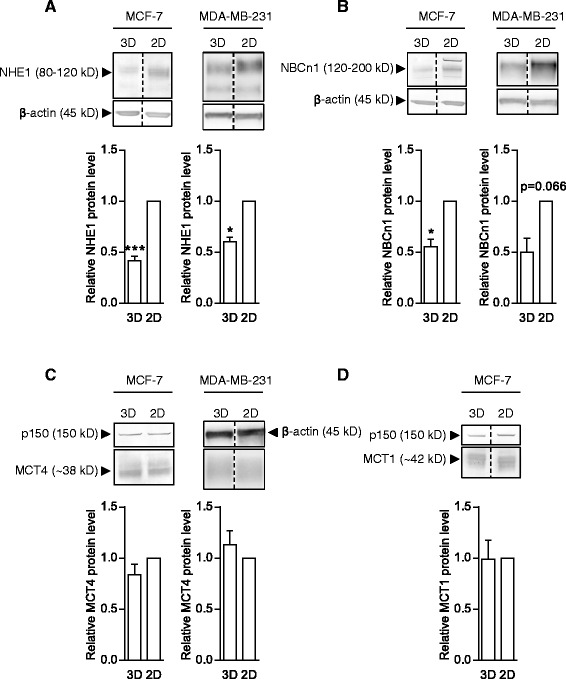
NHE1, NBCn1, MCT1 and MCT4 expression in MCF-7 and MDA-MB-231 spheroids relative to 2D culture. MCF-7 and MDA-MB-231 spheroids (3D) and 2D cultures were grown 4 days in parallel, followed by lysis and Western blotting with antibodies directed against the specific transporters. Top panels in **a**, **b**, **c** and **d** show representative Western blots, while lower panels show quantifications of band intensities normalized to that of corresponding 2D culture. **a**: NHE1 (MCF-7: 5n, MDA-MB-231: 3n). **b**: NBCn1 (MCF-7 and MDA-MB-231: 3n). **c**: MCT4 (MCF-7 and MDA-MB-231: 3n). **d**: MCT1 (3n). Error bars denote SEM. A two-tailed, paired Student’s t-test was used to test for statistically significant difference in means between the two groups. * and *** indicate *p* < 0.05 and *p* < 0.001, respectively

To more precisely compare the transporter distribution through the MCF-7 spheroids to the hypoxia gradient obtained from the pimonidazole analysis shown in Fig. 1e, mean pixel intensity profiles through the spheroids were calculated for each transporter. The summarized profile analyses are shown in Fig. 2h, and individual traces in Additional file 2: Figure S2. As seen, the distribution of MCT1 largely follows the hypoxia gradient, while NHE1 and MCT4 are distributed evenly along the axis, and NBCn1 exhibits the highest expression at the spheroid periphery. To determine whether 3D growth altered the total expression of NHE1, NBCn1, MCT1 and MCT4 compared to 2D growth, MCF-7 and MDA-MB-231 spheroids were lysed and subjected to SDS-PAGE and immunoblotting (Fig. 3). Relative to total protein levels, the expression of NHE1 and NBCn1 was reduced by about 50 % in 3D compared to 2D growth, while that of MCT1 (MCF-7 cells only) and MCT4 was unaltered. Notably, this pattern was identical between the two cell types. Posttranslational regulation of these transporters remains incompletely characterized. The most widely studied is that of Ser703 of human NHE1, which is phosphorylated by the ERK effector p90RSK and is important for NHE1 activation by serum and growth factors [33]. The Ser703 phosphorylation of NHE1, relative to the total NHE1 expression, was not significantly altered by 3D- compared to 2D growth, but was significantly reduced as MDA-MB-231 cells grew in size (Additional file 3: Figure S3).

Collectively, these results show that the four pH-regulatory transporters exhibit distinct patterns of localization in 3D spheroids, and that their relative expression is altered in 3D compared to 2D growth conditions.

### Growth of MCF-7 spheroids is attenuated by inhibition of MCT1/2 and NBCn1

**Fig. 4:**
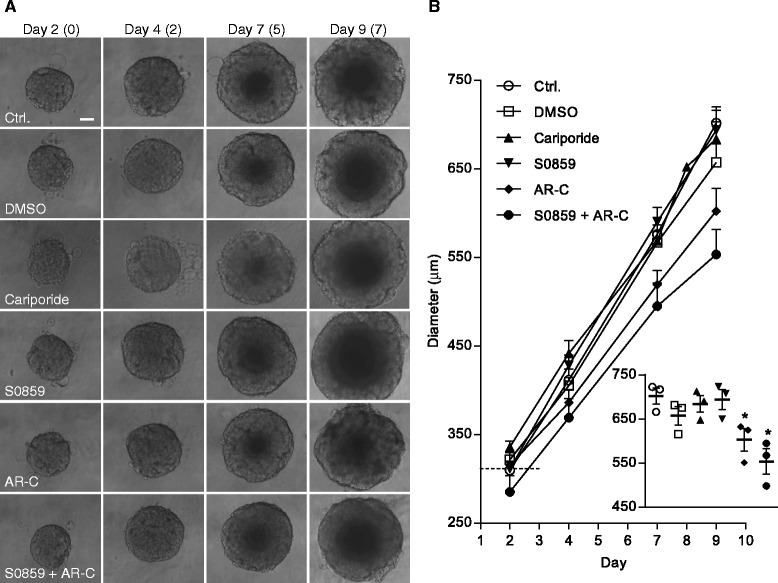
Effect of pharmacological inhibitors of NHE1, NBCn1, and MCT1 on MCF-7 spheroid growth. MCF-7 spheroids were treated with inhibitors of NHE1 (Cariporide, 10 μM), NBC (S0859, 50 μM), and MCT1 (AR-C, 20 μM) on day 2 and their growth was monitored for seven days (until day 9). **a**: Representative light microscopic images of the spheroids (10×) on day 2, 4, 7 and 9. Scalebar: 100 μm. Numbers in parentheses indicate number of days treated with the respective inhibitors. **b**: Quantification of spheroid diameters shown in **a**. *n* = 3-5. Error bars denote SEM. The horizontal dashed line indicates the mean diameter of all spheroids on day 2. There was no statistical significant difference in spheroid diameter between the groups on day 2 (tested by a one-way ANOVA with Tukey’s multiple comparisons post-test). The dotplot insert shows diameters on day 9. Mean and SEM are indicated. One-way ANOVA with Dunnett’s multiple comparisons post-test was used to test for statistically significant differences between treatment groups and their respective vehicle control (Ctrl./DMSO). * indicates *p*-value < 0.05

To pharmacologically determine the importance of the individual transporters in 3D growth of breast cancer cells, MCF-7 cells were grown as 3D spheroids for two days, followed by seven days of growth in the presence of cariporide (10 μM), S0859 (50 μM), and/or AR-C155858 (20 μM) to inhibit NHE1, NBCs, or MCT1/2, respectively (Fig. 4). Control spheroids grew to a diameter of about 700 μm over the 9 days (650 μm in the presence of DMSO as vehicle for AR-C155858). Inhibition of NHE1 by cariporide had no effect on spheroid growth. In contrast, MCT1/2 inhibition significantly reduced day 9 spheroid diameter (Fig. 4b, insert), and combined treatment with AR-C155858 and S0859 further reduced day 9 diameter to about 550 μm, while the NBC inhibitor alone had no effect. In general, no changes in transporter expression relative to the corresponding control conditions were seen after inhibitor treatment (Additional file 4: Figure S4A–C), with the exception that NBCn1 expression was increased when either of the transporters was inhibited, similar to what is seen after NHE1 knockdown [12]. No changes in transporter localization were detectable after treatment with any of the inhibitors (*n* = 2, data not shown). Furthermore, there was no increase in PARP cleavage in AR-C155858- and S0859-treated spheroids compared to control, in fact PARP cleavage was decreased in AR-C treated spheroids (Additional file 4: Figure S4D).

### Knockdown of MCT1 or NBCn1, and complete knockout of NHE1, inhibits growth of MCF-7 spheroids

**Fig. 5:**
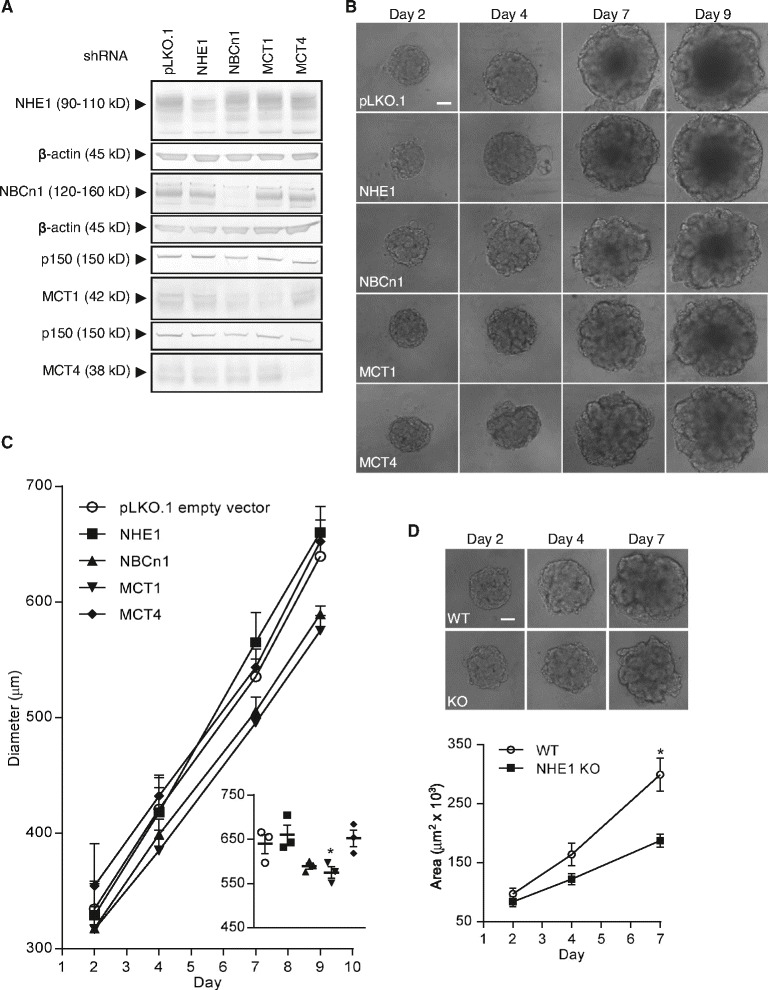
Roles of NHE1, NBCn1, MCT1 and MCT4 in MCF-7 spheroid growth. MCF-7 cells were transduced with lentivirus containing plasmids with shRNA constructs targeted against NHE1, NBCn1, MCT1 or MCT4, respectively. Transduction with the empty vector pLKO.1 (pLKO.1) was used as control. **a**: Western blotting with antibodies directed against the specific transporters was performed to verify knockdown of the respective transporters. **b**: The transduced cell lines were grown as spheroids, and representative light microscopic images (10×) of the spheroids on day 2, 4, 7 and 9 are shown. Scalebar: 100 μm. 3n. **c**: Quantification of spheroid diameters shown in **b**. The dot-plot insert shows diameters on day 9. Mean and SEM are indicated. One-way ANOVA with Dunnett’s multiple comparisons post-test was used to test for statistical significant differences between pLKO.1 and the respective groups. **d**: NHE1 expression was ablated in MCF-7 cells by CRISPR/Cas9-mediated knockout (KO) and wild-type (WT) and KO cells were grown as spheroids for seven days. Lower panel shows quantification of spheroid areas while the top panel shows representative light microscopic images (10×) of the spheroids on day 2, 4 and 7. Scale bar: 100 μm. 3 n. Error bars denote SEM. A Student’s t-test (unpaired) was used to test for statistical significant difference between the wild-type and NHE1 knockout. * indicates *p* < 0.05

While cariporide and AR-C155858 are considered highly specific for NHE1 and MCT1, respectively, there is currently no commercially available specific inhibitor of MCT4, and S0859 was recently reported to also inhibit MCTs [54]. To validate the pharmacological data, each transporter was therefore next stably knocked down in MCF-7 cells, which were then subjected to spheroid growth as above. Knockdown efficiencies were approximately 50, 80, 75 and 80 %, respectively, for NHE1, NBCn1, MCT1 and MCT4 (Fig. 5a). Representative images and summarized growth curves are shown in Fig. 5b–c. As seen, spheroid diameter at day 9 was significantly reduced by knockdown of MCT1, and knockdown of NBCn1 had a numerically similar but not quite statistically significant effect. In contrast, knockdown of NHE1 or MCT4 had no detectable effect on spheroid growth (Fig. 5c). While knockdown of MCT1 or NBCn1 was rather efficient (75–80 %) and inhibited growth, and the effect was similar to that of the corresponding pharmacological agents, NHE1 knockdown was less efficient (50 %), and the lack of effect of NHE1 inhibition or knockdown was puzzling, given the previously reported roles of this transporter in cancer cell proliferation and growth [10, 14]. We therefore performed a full CRISPR/Cas9 knockout of NHE1 in MCF-7 cells (Additional file 5: Figure S5B), and grew these cells as spheroids as above. Indeed, in contrast to the lack of effect of the 50 % knockdown (Fig. 5c), full knockout of NHE1 significantly retarded spheroid growth compared to that of wild-type MCF-7 cells (Fig. 5d).

### Knockdown of NHE1 inhibits growth of MDA-MB-231 spheroids

**Fig. 6:**
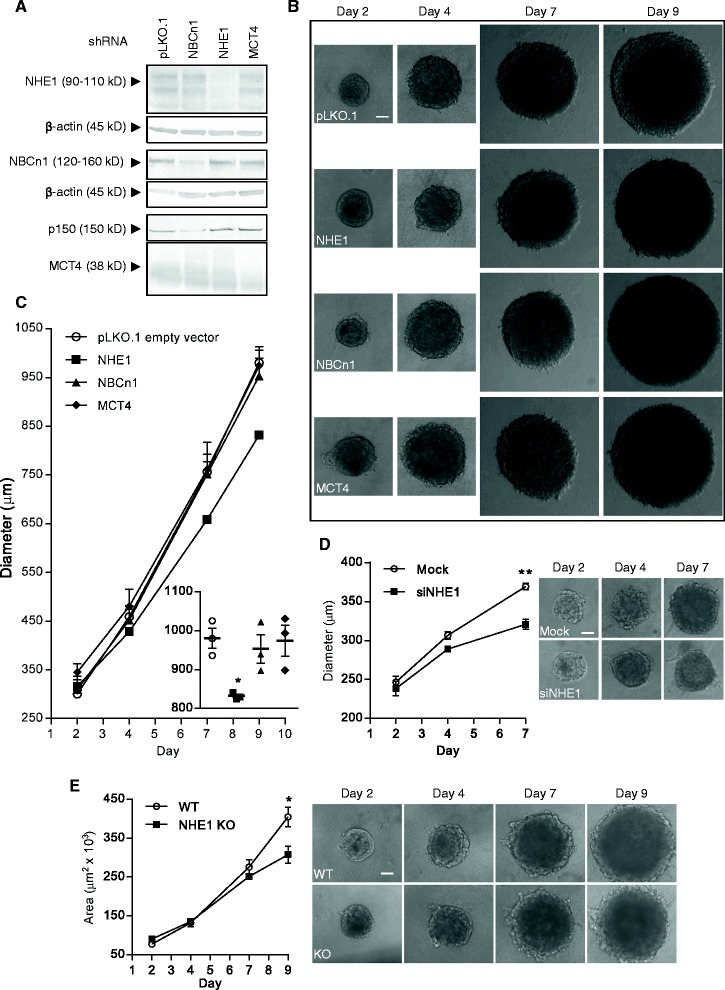
Effect of stable knockdown of NHE1, NBCn1 or MCT4 on growth of MDA-MB-231 spheroids. MDA-MB-231 cells were transduced with lentivirus containing plasmids with shRNA constructs targeted against NHE1, NBCn1 or MCT4. Transduction with the empty vector pLKO.1 (pLKO.1) was used as control. **a**: Western blotting with antibodies directed against the specific transporters was performed to verify knockdown of the respective transporters. **b**: The transduced cell lines were grown as spheroids, and representative light microscopic images (10×) of the spheroids on day 2, 4, 7 and 9 are shown. Scalebar: 100 μm. 3n. **c**: Quantification of spheroid diameters shown in **b**. The dotplot insert shows diameters on day 9. Mean and SEM are indicated. One-way ANOVA with Dunnett’s multiple comparisons post-test was used to test for statistical significant differences between pLKO.1 and the respective groups. **d**: NHE1 was transiently knocked down in MDA-MB-231 cells by siRNA interference and cells were grown as spheroids. Representative light microscopic images (10×) of the spheroids on day 2, 4 and 7 are shown on the right while quantification of spheroid diameters is shown on the left. Scalebar: 100 μm. 3n. Error bars denote SEM. A two-tailed, paired Student’s t-test was used to test for statistically significant difference in means between the two groups on day 7. **e**: NHE1 expression was ablated in MDA-MB-231 cells by CRISPR/Cas9-mediated knockout (KO) and grown as spheroids for nine days. Left panel shows quantification of spheroid areas while the right panel shows representative light microscopic images (10×) of the spheroids on day 2, 4, 7 and 9. Scalebar: 100 μm. 3 n. Error bars denote SEM. WT: wild-type. A two-tailed, unpaired Student’s t-test was used to test for statistically significant difference in means between the two groups on day 9. * and ** indicate *p* < 0.05 and *p* < 0.01, respectively

The activity and expression levels of different pH-regulatory transporters vary widely between different cancer cell types [26, 34, 35]. We therefore next asked whether spheroid growth of another widely used human breast cancer model, the highly invasive MDA-MB-231 cells which represent the triple-negative breast cancer subtype, was affected by stable knockdown of NHE1, NBCn1, or MCT4 (as noted above, MCT1 is not expressed in MDA-MB-231 at detectable levels, [32]). Stable knockdown of all three transporters was obtained, with knockdown efficiencies of about 85, 70 and 70 %, respectively, for NHE1, NBCn1, and MCT4, and no detectable compensatory upregulation of the transporters studied (Fig. 6a). Xenograft tumors grown from these cells were recently shown to be strongly growth-attenuated by NHE1 knockdown [36]. In congruence with this, NHE1 knockdown significantly reduced MDA-MB-231 spheroid diameter at day 9, while knockdown of NBCn1 or MCT4 had no effect (Fig. 6b–c). To further substantiate this finding, we also evaluated the effect of transient knockdown of NHE1 in MDA-MB-231 cells with an unrelated NHE1 siRNA sequence (Fig. 6d; knockdown was stable for at least 120 h, Additional file 5: Figure S5A), and of CRISPR/Cas9 knockout of NHE1 in MDA-MB-231 cells (Fig. 6e, Additional file 5: Figure S5B), both of which significantly reduced spheroid growth. In contrast, addition of cariporide had no effect (Additional file 6: Figure S6; see Discussion).

Collectively, the results in Figs. 4, 5, and 6 show that acid-extruding ion transporters are important for 3D spheroid growth of breast cancer cells. The specific acid extruder(s) involved are cell-type dependent, with MCT1 and NBCn1 playing important roles in MCF-7 cells, and NHE1 apparently playing a greater role in MDA-MB-231 cells, although the more efficient knockdown in these cells should be taken into account.

## Discussion

Acid–base homeostasis is dysregulated in cancer cells and conditions for pH regulation are fundamentally different in 3D compared to 2D environments. Here, we show that localization and/or expression of four major net acid extruding transporters – NHE1, NBCn1, MCT1 and MCT4 – are regulated during growth of breast cancer spheroids and that these transporters contribute to breast cancer spheroid growth in a cell-type dependent manner.

### General properties of MCF-7 spheroids

While in vivo tumors are obviously much more complex than cancer cell spheroids in terms of architectural and cellular diversity, spheroids resemble in vivo tumors much more closely than 2D cultures with respect to multiple parameters, and are excellent models of 3D growth [4, 5]. Normal mammary epithelial cells can be induced to form acini in 3D culture, resembling the native mammary duct structure [29, 31]. With increasing aggressiveness, organization is lost, resulting in the formation of solid spheroids [31, 37] and at larger sizes often a necrotic core [5]. The observation of a central cavity in such a spheroid can thus reflect lumen formation, generally characterized by apoptosis [31] or necrotic core formation due to hypoxia. MCF-7 cell spheroids lack lumen formation after 1–2 days of growth ([29] and APA and SFP, unpublished data), but we show here that continued spheroid growth of MCF-7 cells is associated with partial polarization and formation of a large lumen lined by apical markers and tight junctions, and a progressive increase in PARP cleavage which was most prominent close to the lumen. MDA-MB-231 spheroids were more loosely organized and lacked lumen and detectable PARP cleavage, yet both MCF-7 and MDA-MB-231 cell spheroids exhibited a clear gradient of increasing hypoxia towards the lumen.

### Acid-extruding transporters are differentially distributed in MCF-7 spheroids, and their expression is altered in 3D compared to 2D

In both MCF-7 and MDA-MB-231 cells, expression of NHE1 and NBCn1 relative to total protein was reduced in 3D compared to 2D, while that of MCT1 and MCT4 was unaltered. The precise changes in the 3D setting underlying this difference require further investigation, yet it is well known that the expression of many transcription factors, cytokines, cytoskeletal proteins and other factors potentially relevant to the expression of the transporters studied here, differs profoundly between 2- and 3D conditions [2, 38, 39], underscoring the importance of studying their roles in a 3D setting. Furthermore, whereas NHE1 and MCT4 appeared uniformly distributed throughout the MCF-7 cell spheroids, NBCn1 expression was most prominent in the spheroid periphery and, conversely, MCT1 expression exhibited an inward-directed gradient which largely coincided with the measured gradient of hypoxia. It was also notable that whereas MCT4 expression was essentially fully membrane-localized in MDA-MB-231 cell spheroids, MCT4 was partially localized to intracellular compartment(s) in MCF-7 cells. While not further pursued here, this may reflect differential expression of proteins contributing to MCT4 membrane localization in the two cell lines [40, 41].

To our knowledge, ours is the first study to address how transporter localization is regulated by spheroid growth. In patient breast cancer tissue, we found NHE1 to be most highly expressed in well-perfused, peripheral tumor regions, while NBCn1 expression did not exhibit a detectable spatial gradient [15]. In brain tumors, NHE1 was most highly expressed at the periphery, whereas MCT1 and −4 showed a broader distribution [42]. In xenografts of colorectal and cervical cancer cells, MCT1 was found in the tumor periphery [43]. In our hands, expression of MCT1, but not MCT4, followed the gradient of hypoxia in the spheroids. Functionally, the high MCT1 activity at the hypoxic spheroid core agrees well with the fact that glycolytic metabolism dominates in this region. What upregulates MCT1 expression in this region remains to be elucidated, since the majority of studies on this topic have shown that MCT4, yet not MCT1, is hypoxia-inducible (e.g. [44, 45]). There are however reports of MCT1 upregulation by hypoxia in cancers [46], possibly due to the additional presence of a glucose deprivation gradient [47], a situation also present in spheroids [5]. Interestingly, it was recently reported that while MCT1 expression was unaltered, its activity was increased under hypoxia due to the hypoxic upregulation of CAIX [45].

It is tempting to suggest that the observed distribution of NHE1 and NBCn1 is reflected in different subcellular contributions to pH_i_ regulation and hence to growth, although it should be kept in mind that due to extensive posttranslational regulation, expression levels *per se* say little about transporter function. Ser703 of human NHE1 has been widely implicated in regulation of NHE1 activity [33], and we recently demonstrated its phosphorylation in breast cancer cells in response to prolactin [48]. Relative NHE1 Ser703 phosphorylation was largely unaffected by 3D growth, except for a decrease in MDA-MB-231 cells with time of spheroid growth. Thus, Ser703-dependent NHE1 activity may be reduced in MDA-MB-231 spheroids during long-term growth. NHE1 was previously found to play a major functional role in pH_i_ regulation in the periphery of cancer spheroids, and HCO_3_^−^ dependent mechanisms in the core [26]. However, the role of HCO_3_^−^ in the core at least in part reflects its role as a mobile buffer, rather than as a substrate for Na^+^,HCO_3_^−^ cotransporters [26]. Given the importance of NBCn1 in breast cancer [12, 15, 22] we focus on this isoform here. A full analysis of all HCO_3_^−^ transporters is beyond the scope of this work, but would be needed to precisely map their contributions and activity, but roles of other isoforms are clearly also likely ([53] and discussion below). Similar to MCT1, the mechanisms causing NBCn1 to be most strongly expressed in the spheroid periphery remain to be determined, but likely regulators would be the gradients of hypoxia, lactate, pH_e_, pH_i_, and ATP arising in spheroids [5].

It should be noted that other acid–base transporters than the four studied here may play a role in 3D growth, depending on the cell type and conditions. For instance, pharmacological inhibitors or knockdown of proton ATPases have been shown to reduce growth of some cancer cells [49], and such compounds are currently in clinical trials [50]. Finally, while not further studied here, it is worth noting that the marked upregulation of CAIX as well as its specific localization to the inner regions of the spheroids, may also be important for the regulation of spheroid growth, given its known importance for pH homeostasis in the confined 3D space of spheroids [51].

### Growth of breast cancer spheroids is dependent on acid extruding ion transport proteins

A major conclusion of this work is that acid-extruding transporter(s) are important for spheroid growth yet that the specific transporters that play the predominant roles differ between breast cancer subtypes. This suggests that what is required to maintain 3D growth is the phenotype of acid extrusion rather than a given transporter protein, posing the challenge to therapeutic use that the relevant target(s) will likely differ between breast cancer subtypes, a notion corroborated by the differences between MCF-7- (luminal A) and MDA-MB-231 (triple-negative) cell spheroids revealed by the present work. Although complete knockout of NHE1 reduced spheroid growth in both cell lines, partial knockdown of NHE1 only reduced growth for MDA-MB-231 spheroids, and growth of MCF-7 spheroids was also delayed by knockdown of NBCn1 or MCT1 or by pharmacological inhibition of MCT, exacerbated by concomitant inhibition of NBCs. The role of MCT1 in spheroid growth is well in line with previous reports from in vivo studies of tumor growth [13, 16, 21]. The role for NBCn1 corroborates previous reports from us and others demonstrating its upregulation in human breast cancer patients [15] and the importance of NBCs in mammary tumor pH_i_ regulation and in vivo tumor formation [14, 22]. In conjunction with GWAS reports linking NBCn1 to breast cancer risk [20], this identifies NBCn1 as a target of potential therapeutic interest. However, the very marked differences in expression of the various NBC isoforms across different cancers [52] suggests that the specific NBC isoform relevant is likely to differ, a notion substantiated by the recently reported role of another SLC4 family member, NBCe1 (SLC4A4), in proliferation of MDA-MB-231 cells as well as LS174 colon cancer cells [53]. In congruence with our finding that NBCn1 expression did not follow the hypoxia gradient in the spheroids, this study furthermore showed that NBCe1, but not NBCn1, was upregulated by hypoxia [53]. Importantly, the compound used to inhibit NBC activity, S0859, was recently shown to also inhibit MCTs [54]. Since in our work, this compound had no effect on its own, but was additive to the effect of the MCT1 inhibitor, we favor the interpretation that NBCn1 is the main target of inhibition in our setup. This was confirmed by the knockdown data, however, a slight reduction in MCT1 expression was seen after NBCn1 knockdown (Fig. **5**a), hence we cannot fully exclude a contribution from MCT1 to the observed effect.

In contrast to MCF-7 cell spheroids, MDA-MB-231 spheroids were not dependent on NBCn1 for growth, but depended only on NHE1 of the transporters studied here. This is supported by early experiments on xenograft growth of human bladder carcinoma cells [55], and recent work demonstrating that NHE1 ablation in MDA-MB-231 cells reduces xenograft growth [36]. Dependence on NHE1 may in part relate to glycolysis status: 50 % of tumors of CCL39 cells inoculated into nude mice underwent spontaneous regression if lacking NHE1 [21], yet growth of non-glycolytic CCL39 cell tumors was unaffected by the absence of NHE1 [56]. In line with this, MDA-MB-231 cells are more dependent on glycolysis than MCF-7 cells [57]. It is furthermore intriguing that NHE1 has been proposed to be particularly dependent on glycolytically derived ATP [58], suggesting that the link between metabolic profile and NHE1 dependence should be further explored.

Finally, despite the marked effects of transporter knockdown or knockout, pharmacological inhibition had no (NHE1) or limited (NBCn1) effect. The same concentration of cariporide strongly attenuated growth of BxPC-3 pancreatic spheroids (Noehr-Nielsen, A., and SFP, unpublished), and although S0859 is very lipophilic [59], the concentration used was previously found effective in spheroids [26] and indeed was additive to that of MCT1 inhibition in the present study. Hence, while they are likely less effective in spheroids than in 2D conditions, it seems unlikely that the inhibitors were not functional. An obvious difference between pharmacological inhibition and knockdown in the present work is that the inhibitors were only present from day 2 after spheroid formation. However, spheroids of knockdown cells were similar in size to controls at this time, hence, elucidation of this point requires further analysis.

We did not detect obvious changes in the core/lumen area in S0859- or AR-C-treated spheroids (*n* = 2, data not shown), and there was no detectable increase in PARP cleavage in these spheroids compared to control, hence, although this remains to be directly addressed, we favor the interpretation that the decrease in spheroid size mainly reflects reduced growth/proliferation. Complete elucidation of the relation between pH_i_ regulation and 3D growth requires further studies. While pH_i_ recovery after an acid load in 2D-grown MCF-7 cells was dependent on both NHE1 and NBCs [12], 3D growth of MCF-7 cells appeared to be more strongly dependent on NBCn1, and only full ablation of NHE1 reduced their growth. One interpretation of this is that hypoxia and strong extracellular acidity in the 3D setting limits contributions from NHE1 to pH_i_ regulation [34], limiting its role in growth at least in the MCF-7 spheroids.

Our work thus corroborates and extends previous work pointing to the therapeutic potential of inhibiting acid extruding transporters in breast cancer. However, several open questions and challenges remain. It is noteworthy that the impact of NBCn1 knockdown on spheroid growth appears less dramatic than the strong inhibitory effect of NBCn1 knockout on growth of chemically induced tumors in vivo [22], and the same appears to be true for NHE1 knockdown, the effect of which on spheroid growth of MDA-MB-231 cells appears to be smaller than that on their xenograft growth in vivo [36]. This raises the exciting possibility that the role(s) of the transporters involves additional environmental factors present in vivo, a question which should be further addressed in future studies. A challenge is the limited specificity of some available pharmacological tools, especially problematic for NBCn1, for which currently available drugs are unspecific and/or unsuitable for tissue use (see [14, 54, 59]). A second challenge illustrated by the present findings is to determine the relevant transporter(s) to target in a given cancer, and under which conditions. Clearly, transporter inhibition is likely to be most effective in combination with other therapeutic modalities, as previously suggested by findings by us and others [12, 36, 60].

## Conclusions

We show here that 3D spheroid growth of MCF-7 breast cancer cells is associated with polarization, hypoxia gradient induction, and changes in the relative expression of acid extruding ion transporters NHE1 and NBCn1 relative to 2D growth. Further, we show that individual pH-regulatory transporters exhibit distinct and differential expression profiles in breast cancer cell spheroids, with MCT1 expression following the hypoxia gradient. Growth of MCF-7 spheroids was predominantly dependent on MCT1 and NBCs/NBCn1 but was also reduced by complete NHE1 knockout, whereas growth of MDA-MB-231 spheroids was predominantly dependent on NHE1.

## Abbreviations

3D, 3-dimensional; AR-C: AR-C155858; ANOVA, Analysis of Variance; ATP, Adenosine triphosphate; CAIX, Carbonic Anhydrase; DMA, Dimethylamiloride; DMSO, Dimethyl sulfoxide; ERM, Ezrin/Radixin/Moesin; GWAS, Genome-wide association study; HE, Hematoxylin-eosin; MCT1/4, Monocarboxylate transporter 1/4; NBCn1, Na^+^,HCO_3_^−^ cotransporter 1; NHE1, Na^+^/H^+^ exchanger 1; PARP-1, Poly (ADP-ribose) polymerase; PBS, Phosphate-buffered saline; pH_e_, extracellular pH; pH_i_, Intracellular pH; pRb, phospho-Retinoblastoma; RT, room temperature; SDS, Sodium dodecyl sulfate; SEM, Standard Error of Mean; SNP, Single Nucleotide Polymorphism; TBST, Tris-buffered saline-Tween; uPA, Urokinase-type plasminogen activator

## Additional files

Additional file 1: Figure S1. PARP cleavage and CAIX expression on day 4 and 9 in MCF-7 and MDA-MB-231 spheroids. A: MCF-7 spheroids grown for 4 and 9 days, respectively, followed by lysis and Western blotting with PARP antibodies. Top panel shows a representative Western blots, while lower panel shows quantifications of band intensities normalized to that of spheroids harvested on day 4. Error bars denote SEM. 3n. A two-tailed, paired Student’s t-test was used to test for statistically significant difference in means between the two groups. B: MCF-7 (i) and MDA-MB-231 (ii) spheroids grown for 9 days followed by immunohistochemical staining with antibodies recognizing cleaved PARP (c-PARP) only. Scalebar: 100 μm. C: MCF-7 (i) and MDA-MB-231 (ii) spheroids (3D) and 2D cultures grown for 4 and 9 days (spheroids only), respectively, in parallel followed by lysis and Western blotting with CAIX antibodies. Blots are representative of 3n. In C_ii_, right panel shows quantifications of band intensities normalized to that of spheroids harvested on day 4. Error bars denote SEM. A two-tailed, paired Student’s t-test was used to test for statistically significant difference in means between the two groups. ns and * indicate non-significant and *p* < 0.05, respectively. (PDF 343 kb) Additional file 2: Figure S2. Transporter distribution across MCF-7 spheroids (individual plots for the data summarized in Fig. 2h). A, B, C, D and E: Relative distribution of the transporters NHE1, NBCn1, MCT1 and −4, and Pimonidazole (a marker of hypoxia), respectively, from the periphery towards the core (across the viable region) of MCF-7 spheroids. Two mean pixel intensity profiles were made per spheroid, on a total of two-three spheroids from independent biological replicates per transporter/antibody, using ImageJ software. Intensity profiles were plotted (black dotted lines) and LOWESS curves with 20 points in the smoothing window (red lines) were drawn using Graphpad PRISM 6. (PDF 149 kb) Additional file 3: Figure S3. NHE1 Ser703 phosphorylation in spheroids and 2D culture and during spheroid growth. MCF-7 and MDA-MB-231 spheroids (3D) and 2D cultures were grown 4 and 9 (MDA-MB-231 spheroids only) days in parallel, followed by lysis and Western blotting with antibodies directed against pSer703-NHE1 and total NHE1. Left and top panels in A and B, respectively, show representative Western blots, while right and lower panels, respectively, show quantifications of band intensities normalized to that of corresponding 2D or 3D culture on day 4. A: MCF-7. Note that the level of pSer703-NHE1 was normalized to the mean total NHE1 level from five other experiments (the total NHE1 data shown in Fig. 3a). Data is shown as mean + SD. 2n. B: MDA-MB-231. Data is shown as mean + SEM. 3n. A two-tailed, paired Student’s t-test was used to test for statistically significant difference in means between two groups. * indicates *p* < 0.05. (PDF 141 kb) Additional file 4: Figure S4. Effects of pharmacological inhibitors on transporter expression in 2- and 3D culture. Top panels in A, B, C and D show representative Western blots of MCF-7 spheroid (3D) and 2D cultures, respectively, treated with S0859 (50 μM) and AR-C (20 μM) for 2 days/48 h. Lower panels show quantifications of band intensities normalized to corresponding vehicle control (Ctrl. or DMSO). Data is shown as mean + SEM. A: NHE1 (4-6n), B: NBCn1 (3n), C: MCT-1 (3n), D: PARP (3n). One-way ANOVA with Dunnett’s multiple comparisons post-test was used to test for statistical significant differences between the control and the respective groups. * and ** indicate p-value < 0.05 and p-value < 0.01, respectively. (PDF 250 kb) Additional file 5: Figure S5. Stability of transient siRNA-mediated knockdown of NHE1 in MDA-MB-231 cells. A. MDA-MB-231 cells were transfected with 100 nM siNHE1. 48 h after transfection, cells were reseeded and the stability of the knockdown in 2D culture was monitored for 120 h. Left panel show Western blots and right panel show quantifications of band intensities normalized to corresponding Mock control. B: Western blots showing CRISPR/Cas9-mediated knockout (KO) of NHE1 in MCF-7 and MDA-MB-231 cells. WT: wild-type. (PDF 135 kb) Additional file 6: Figure S6. Effect of cariporide on MDA-MB-231 spheroid growth. MDA-MB-231 spheroids were treated with Cariporide (10 μM) on day 2 and their growth was monitored for seven days (until day 9). A: Representative light microscopic images (10×) of the spheroids on day 2, 4, 7, and 9. Numbers in parentheses indicate number of days treated with the respective inhibitors. Scalebar: 100 μm. B: Quantification of spheroid diameters shown in A. *n* = 2-3. Error bars denote SEM. (PDF 275 kb)

## Footnotes

1 Part of these findings have previously been reported in abstract form [27].
