# Supplementary figures and images for: Roles of acid-extruding ion transporters in regulation of breast cancer cell growth in a 3-dimensional microenvironment
Source: Mol Cancer. 2016 Jun 6;15:45. doi: 10.1186/s12943-016-0528-0 (PMC4896021; doi:10.1186/s12943-016-0528-0)

**Figure S1 (Andersen et al.)**

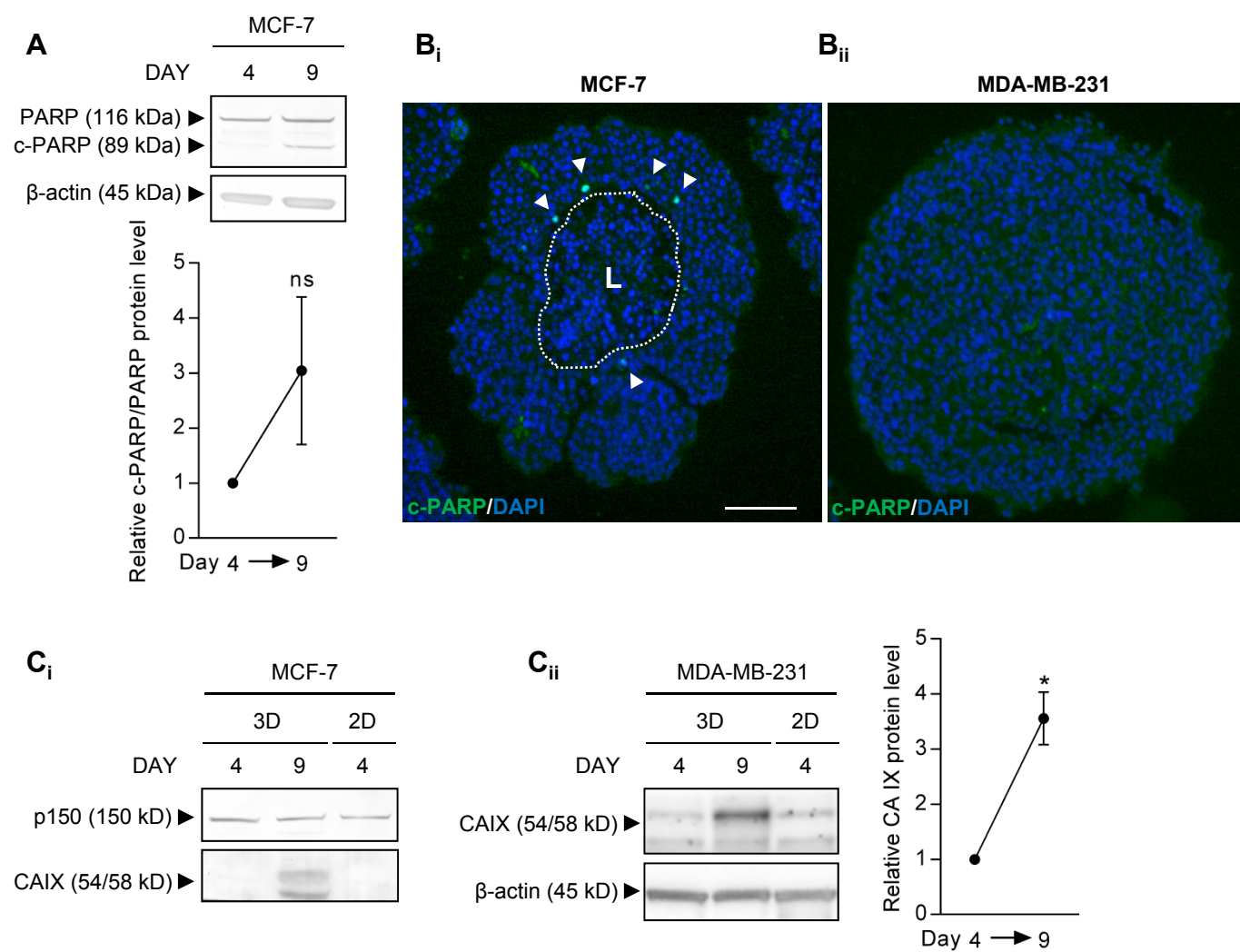

Supplement: Additional file 1: Figure S1. — PARP cleavage and CAIX expression on day 4 and 9 in MCF-7 and MDA-MB-231 spheroids. A: MCF-7 spheroids grown for 4 and 9 days, respectively, followed by lysis and Western blotting with PARP antibodies. Top panel shows a representative Western blots, while lower panel shows quantifications of band intensities normalized to that of spheroids harvested on day 4. Error bars denote SEM. 3n. A two-tailed, paired Student’s t-test was used to test for statistically significant difference in means between the two groups. B: MCF-7 (i) and MDA-MB-231 (ii) spheroids grown for 9 days followed by immunohistochemical staining with antibodies recognizing cleaved PARP (c-PARP) only. Scalebar: 100 μm. C: MCF-7 (i) and MDA-MB-231 (ii) spheroids (3D) and 2D cultures grown for 4 and 9 days (spheroids only), respectively, in parallel followed by lysis and Western blotting with CAIX antibodies. Blots are representative of 3n. In Cii, right panel shows quantifications of band intensities normalized to that of spheroids harvested on day 4. Error bars denote SEM. A two-tailed, paired Student’s t-test was used to test for statistically significant difference in means between the two groups. ns and * indicate non-significant and p < 0.05, respectively. (PDF 343 kb) [file 12943_2016_528_MOESM1_ESM.pdf]

Figure S2 (Andersen et al.)

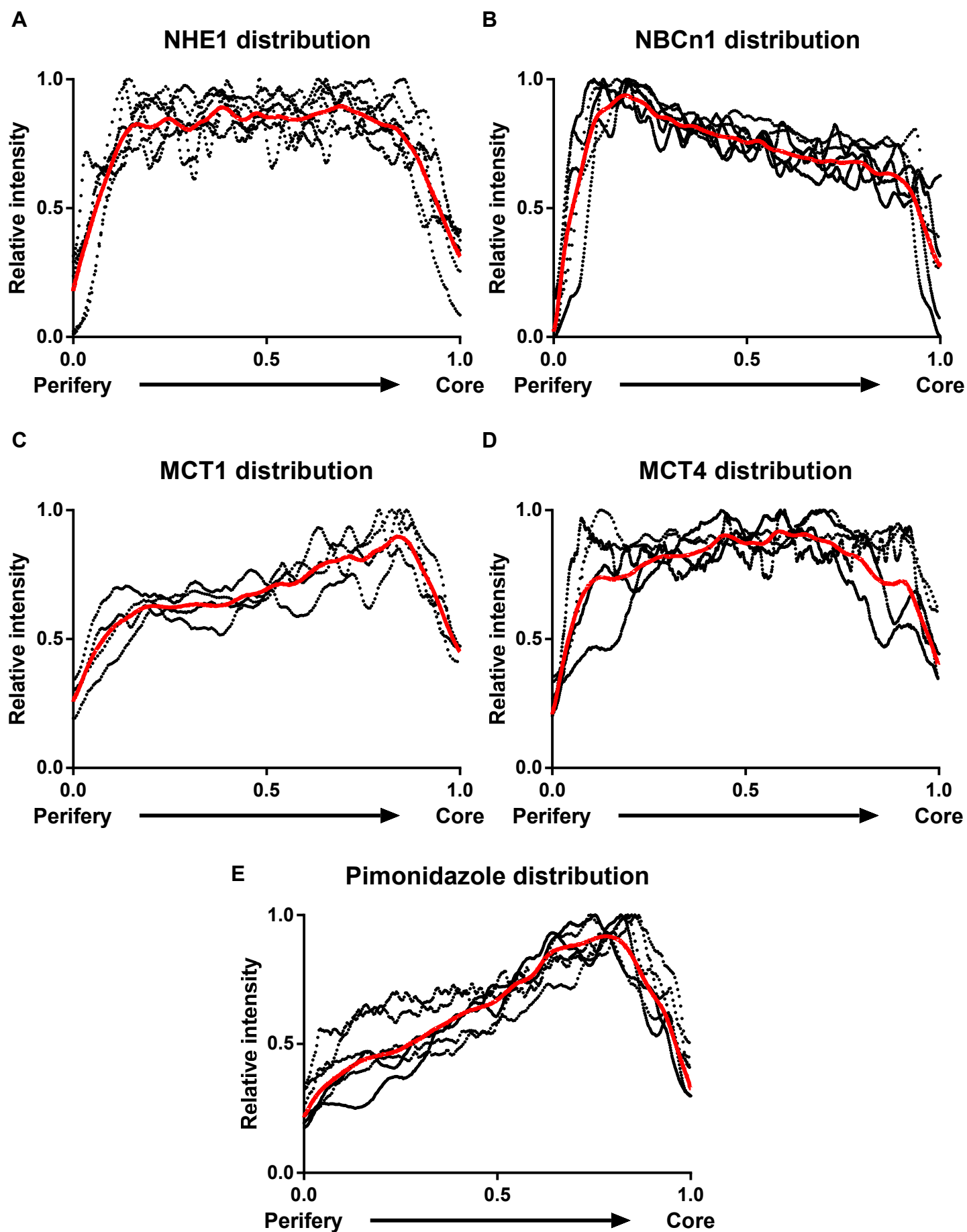

Supplement: Additional file 2: Figure S2. — Transporter distribution across MCF-7 spheroids (individual plots for the data summarized in Fig. 2h). A, B, C, D and E: Relative distribution of the transporters NHE1, NBCn1, MCT1 and −4, and Pimonidazole (a marker of hypoxia), respectively, from the periphery towards the core (across the viable region) of MCF-7 spheroids. Two mean pixel intensity profiles were made per spheroid, on a total of two-three spheroids from independent biological replicates per transporter/antibody, using ImageJ software. Intensity profiles were plotted (black dotted lines) and LOWESS curves with 20 points in the smoothing window (red lines) were drawn using Graphpad PRISM 6. (PDF 149 kb) [file 12943_2016_528_MOESM2_ESM.pdf]

**Figure S3 (Andersen et al.)**

**A**

**MCF-7**

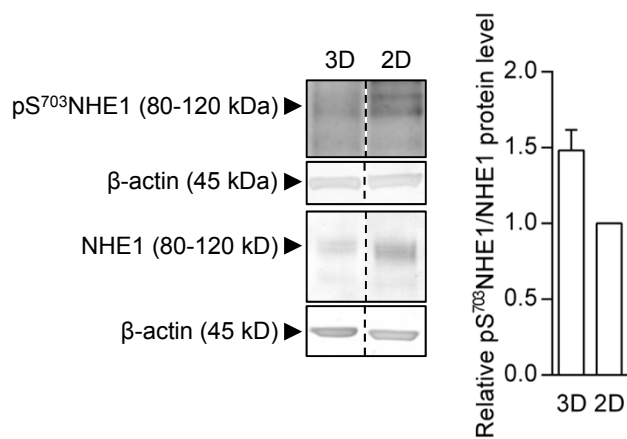

**B**

**MDA-MB-231**

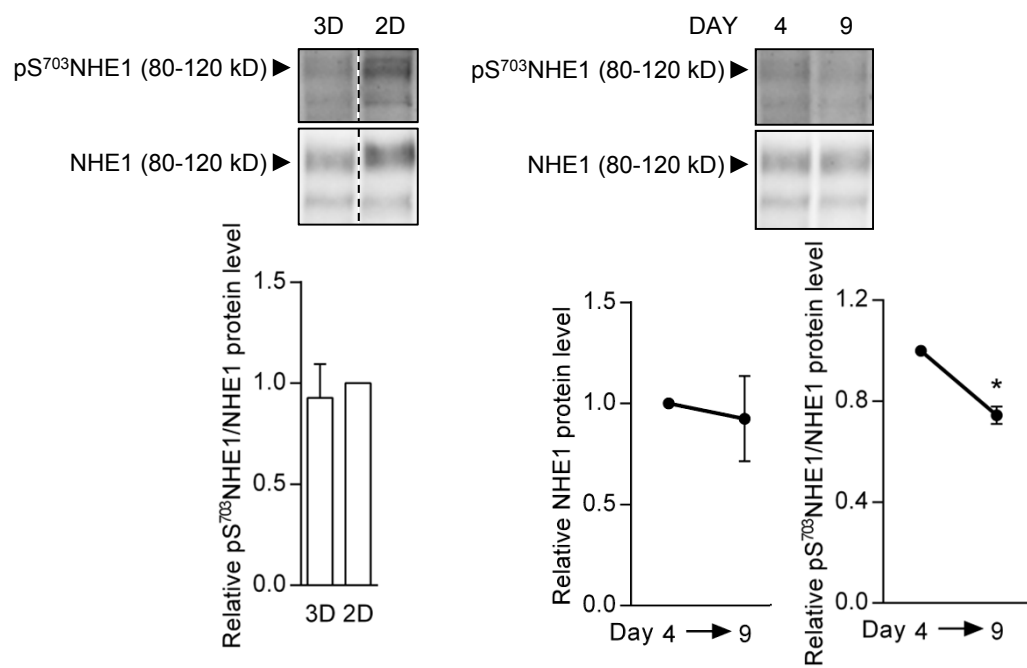

Supplement: Additional file 3: Figure S3. — NHE1 Ser703 phosphorylation in spheroids and 2D culture and during spheroid growth. MCF-7 and MDA-MB-231 spheroids (3D) and 2D cultures were grown 4 and 9 (MDA-MB-231 spheroids only) days in parallel, followed by lysis and Western blotting with antibodies directed against pSer703-NHE1 and total NHE1. Left and top panels in A and B, respectively, show representative Western blots, while right and lower panels, respectively, show quantifications of band intensities normalized to that of corresponding 2D or 3D culture on day 4. A: MCF-7. Note that the level of pSer703-NHE1 was normalized to the mean total NHE1 level from five other experiments (the total NHE1 data shown in Fig. 3a). Data is shown as mean + SD. 2n. B: MDA-MB-231. Data is shown as mean + SEM. 3n. A two-tailed, paired Student’s t-test was used to test for statistically significant difference in means between two groups. * indicates p < 0.05. (PDF 141 kb) [file 12943_2016_528_MOESM3_ESM.pdf]

**Figure S4 (Andersen et al. )**

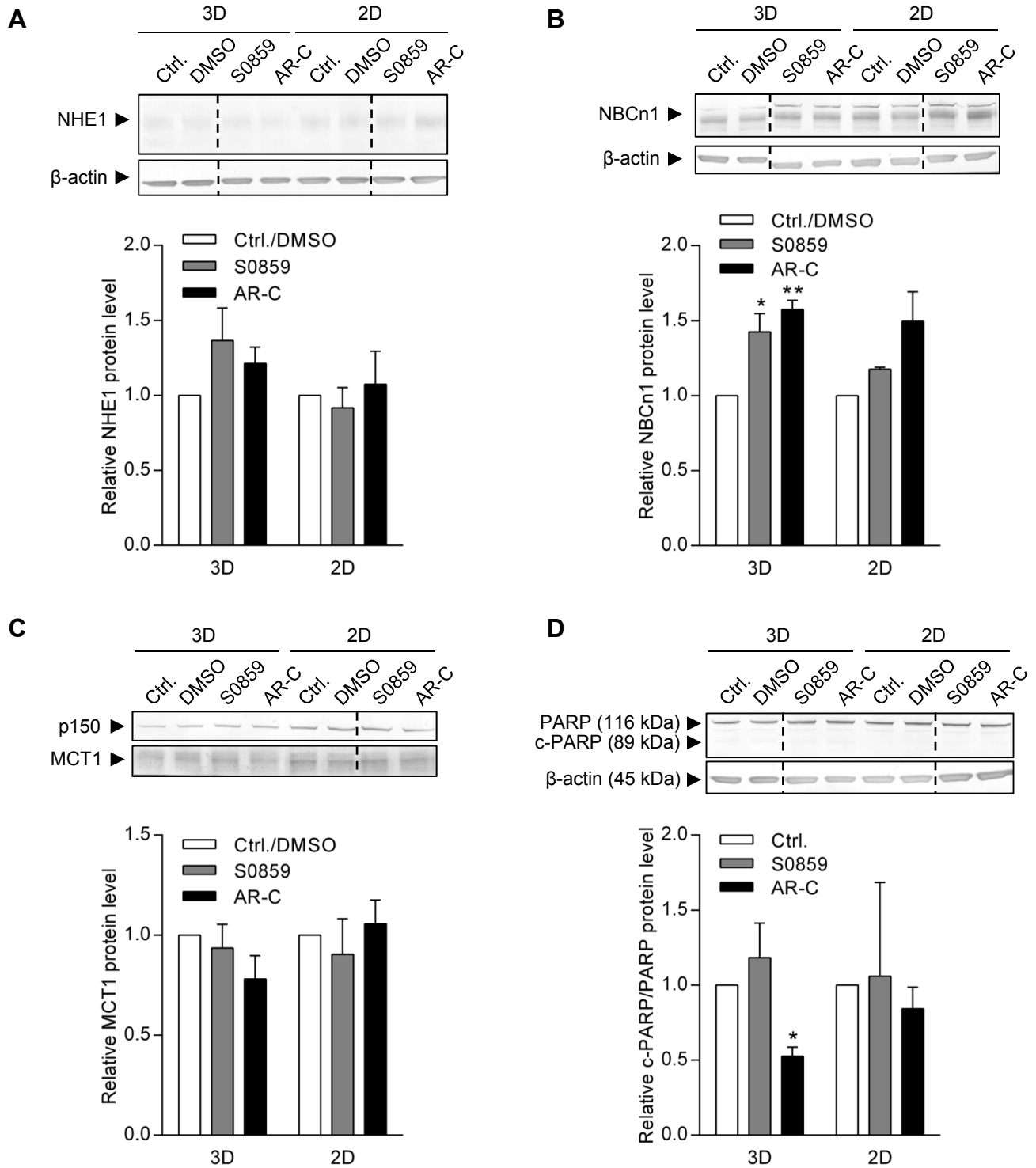

Supplement: Additional file 4: Figure S4. — Effects of pharmacological inhibitors on transporter expression in 2- and 3D culture. Top panels in A, B, C and D show representative Western blots of MCF-7 spheroid (3D) and 2D cultures, respectively, treated with S0859 (50 μM) and AR-C (20 μM) for 2 days/48 h. Lower panels show quantifications of band intensities normalized to corresponding vehicle control (Ctrl. or DMSO). Data is shown as mean + SEM. A: NHE1 (4-6n), B: NBCn1 (3n), C: MCT-1 (3n), D: PARP (3n). One-way ANOVA with Dunnett’s multiple comparisons post-test was used to test for statistical significant differences between the control and the respective groups. * and ** indicate p-value < 0.05 and p-value < 0.01, respectively. (PDF 250 kb) [file 12943_2016_528_MOESM4_ESM.pdf]

**Figure S5 (Andersen et al.)**

**A**

**MDA-MB-231**

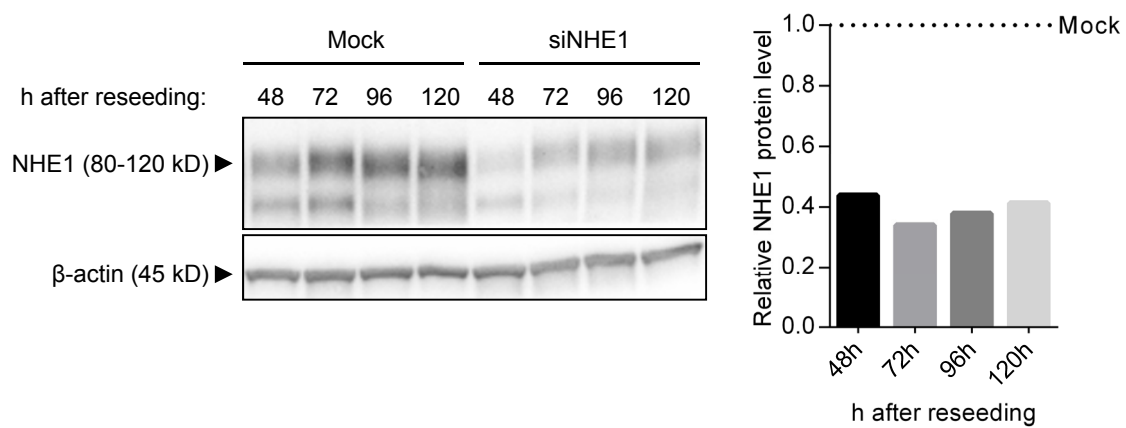

**B**

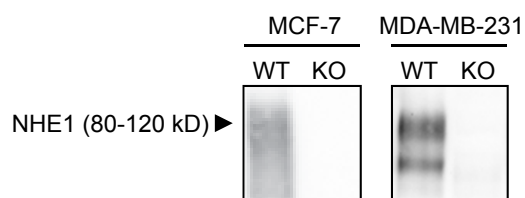

Supplement: Additional file 5: Figure S5. — Stability of transient siRNA-mediated knockdown of NHE1 in MDA-MB-231 cells. A. MDA-MB-231 cells were transfected with 100 nM siNHE1. 48 h after transfection, cells were reseeded and the stability of the knockdown in 2D culture was monitored for 120 h. Left panel show Western blots and right panel show quantifications of band intensities normalized to corresponding Mock control. B: Western blots showing CRISPR/Cas9-mediated knockout (KO) of NHE1 in MCF-7 and MDA-MB-231 cells. WT: wild-type. (PDF 135 kb) [file 12943_2016_528_MOESM5_ESM.pdf]

**Figure S6 (Andersen et al.)**

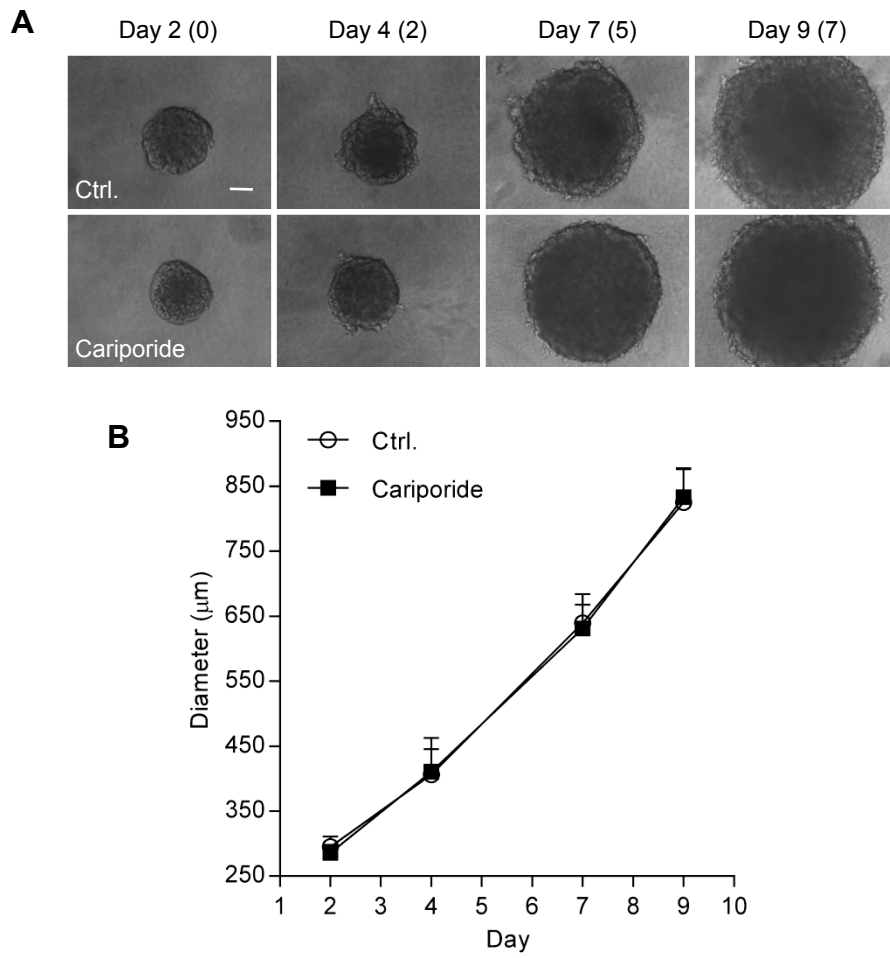

Supplement: Additional file 6: Figure S6. — Effect of cariporide on MDA-MB-231 spheroid growth. MDA-MB-231 spheroids were treated with Cariporide (10 μM) on day 2 and their growth was monitored for seven days (until day 9). A: Representative light microscopic images (10×) of the spheroids on day 2, 4, 7, and 9. Numbers in parentheses indicate number of days treated with the respective inhibitors. Scalebar: 100 μm. B: Quantification of spheroid diameters shown in A. n = 2-3. Error bars denote SEM. (PDF 275 kb) [file 12943_2016_528_MOESM6_ESM.pdf]
